# *Mycoplasma genitalium*: An Emerging Cause of Pelvic Inflammatory Disease

**DOI:** 10.1155/2011/959816

**Published:** 2011-12-25

**Authors:** Catherine L. Haggerty, Brandie D. Taylor

**Affiliations:** ^1^Department of Epidemiology, University of Pittsburgh, Pittsburgh, PA 15261, USA; ^2^Department of Epidemiology, Michigan State University, East Lansing, MI 48824, USA

## Abstract

*Mycoplasma genitalium* is a sexually transmitted pathogen that is increasingly identified among women with pelvic inflammatory disease (PID). Although *Chlamydia trachomatis* and *Neisseria gonorrhoeae* frequently cause PID, up to 70% of cases have an unidentified etiology. This paper summarizes evidence linking *M. genitalium* to PID and its long-term reproductive sequelae. Several PCR studies have demonstrated that *M. genitalium* is associated with PID, independent of gonococcal and chlamydial infection. Most have been cross-sectional, although one prospective investigation suggested that *M. genitalium* was associated with over a thirteenfold risk of endometritis. Further, a nested case-control posttermination study demonstrated a sixfold increased risk of PID among *M. genitalium* positive patients. Whether or not *M. genitalium* upper genital tract infection results in long-term reproductive morbidity is unclear, although tubal factor infertility patients have been found to have elevated *M. genitalium* antibodies. Several lines of evidence suggest that *M. genitalium* is likely resistant to many frequently used PID treatment regimens. Correspondingly, *M. genitalium* has been associated with treatment failure following cefoxitin and doxycycline treatment for clinically suspected PID. Collectively, strong evidence suggests that *M. genitalium* is associated with PID. Further study of *M. genitalium* upper genital tract infection diagnosis, treatment and long-term sequelae is warranted.

## 1. Introduction

*Mycoplasma genitalium* is a genital tract microorganism [1, 2] identified in approximately 15 to 20% of young women seen in some adolescent health centers, sexually transmitted infection clinics, and emergency departments in the United States [3–6]. Concordance of *M. genitalium* infection [1, 2, 7, 8] as well as *M. genitalium* sequence type [9] among sexual partners suggests that this bacteria is sexually transmitted. In some populations studied, infection with *M. genitalium* is as common as *Chlamydia trachomatis* among high risk sexually active women [3, 10] and women with clinically suspected pelvic inflammatory disease (PID) [4]. As *C. trachomatis* is the most common reportable bacterial infection in the United States [11], *M. genitalium* is thus a relatively common infection. *M. genitalium* has been associated with cervicitis [2, 12–15] and may play a role in PID, the infection and inflammation of a woman's upper genital tract [16]. 

PID is frequent among women of childbearing age, diagnosed in approximately 8% of US women and 15% of Swedish women in their lifetime, with over one million U.S. women treated annually [17–22]. Major reproductive and gynecologic morbidities result from PID, including infertility, ectopic pregnancy, chronic pelvic pain, and recurrent PID [23]. Although PID has a polymicrobial etiology, with *C. trachomatis* and/or *N. gonorrhoeae* isolated from approximately one-third to one half of cases [5, 24–27], many PID cases have an unidentified etiology. Although bacterial vaginosis-associated and mycoplasmal organisms have been associated with PID [4–6, 13, 25, 27–32], independent of gonococcal and chlamydial infection [4, 28], less is known about the etiology, treatment, and sequelae of nongonococcal, nonchlamydial PID. This paper reviews recent evidence for the role of *M. genitalium* in PID and subsequent reproductive and gynecologic outcomes.

## 2. *Mycoplasma genitalium* Lower Genital Tract Infection

*M. genitalium* was first identified in the early 1980s among men with nongonococcal urethritis [33]. Because the microbe is extremely difficult to culture, only with polymerase chain reaction (PCR) technology has research into the pathogenicity of *M. genitalium* progressed. Numerous studies have confirmed the role of *M. genitalium* in acute and chronic drug-resistant nongonococcal urethritis [34–36]. In women, *M. genitalium* has been positively associated with cervical inflammation and clinically diagnosed cervicitis, although variable case definitions of cervicitis are responsible for some discrepancies in this literature [12]. As *C. trachomatis *is a common cause of cervicitis and thus may confound this series of studies, some have excluded patients testing positive for *C. trachomatis* or have adjusted for it in multivariate analyses. The vast majority of these have demonstrated an independent, significant association between *M. genitalium* and cervicitis [12].

## 3. *Mycoplasma genitalium* and PID

PID typically occurs as microorganisms ascend from the lower genital tract and through the cervical os, infecting the uterus, fallopian tubes, and ovaries. Thus, cervicitis is a common antecedent of PID. Because *M. genitalium* is associated with cervicitis [2, 13–15], it is reasonable that it also causes nongonococcal, nonchlamydial PID. Indeed, this organism induces salpingitis in monkeys [37, 38], has been found to ascend from the lower to the upper genital tract in a mouse model [39], causes morphologic changes in ciliated fallopian tube cells in vitro [40], and has been detected in fallopian tube tissue in a woman with salpingitis [41]. Further, *M. genitalium* has been shown to adhere to human spermatozoa, and therefore may potentially be carried by motile sperm to the female upper genital tract [42]. 

*M. genitalium* is detected by PCR frequently among women with PID, with rates ranging from 13% to 16% [4, 6, 43]. Several epidemiologic studies have associated *M. genitalium* with clinically suspected PID, endometritis, and adnexitis (see Table 1) [4, 6, 13, 32, 41, 43, 45]. In particular, a handful of studies have examined the relationship between *M. genitalium* identified by PCR and either histologically confirmed endometritis or salpingitis among a population of women with clinically suspected PID [4, 6]. In a study of 115 women presenting to a sexually transmitted disease clinic in Nairobi, Kenya, women with histologically confirmed endometritis were significantly more likely to have *M. genitalium* identified by PCR from the cervix and/or endometrium (16% versus 2%, *P* = 0.02) [6]. After excluding women with gonococcal or chlamydial infection, this study demonstrated an independent association between *M. genitalium* and PID [6]. Similarly, in the PEACH study, Haggerty et al. reported that 15% (88) of 586 women with clinically suspected PID tested positive for *M. genitalium* in the cervix and/or endometrium by PCR. These women were more than twice as likely to have histologically confirmed endometritis at baseline (OR 2.6, 95% CI 1.5–4.6) as compared to women without *M. genitalium* identified at either site, and this relationship remained significant after adjustment for age, race, and gonococcal and chlamydial infection (adjusted OR 2.0, 95% CI 1.0–4.2) [4]. 

A weakness of the above investigations and a problem which challenges many PID studies are the lack of a true comparison group without signs and symptoms of PID. That is, the control groups were comprised of women with clinically suspected PID who did not have histologically confirmed endometritis. In an attempt to overcome this limitation, a few studies have been conducted with control groups comprised of women without clinically suspected PID. In a study of 53 patients with PID and 80 asymptomatic pregnant women recruited from an obstetrics and gynecology clinic, Uno et al. demonstrated a higher prevalence of *M. genitalium* detected by PCR among the women with PID as compared to controls (6% versus 0%) [13]. In another study of 45 patients with clinically suspected PID and 37 control women undergoing tubal ligation, *M. genitalium* was detected by PCR in 13% of cases versus 0% of controls [43]. These studies collectively demonstrate a higher prevalence of *M. genitalium* among PID patients as compared to external controls, but are limited by the lack of upper genital tract sampling. One study of 194 patients with clinically suspected PID and 246 asymptomatic pregnant women being screened for rubella compared the seroprevalence of *M. genitalium* using a lipid-associated membrane protein-enzyme immunoassay (LAMP-EIA) [44]. Before and after adjustment for chlamydial antibodies, *M. genitalium* was not associated with PID (OR 1.0, 95% CI 0.6–1.7). The null association may be explained by the use of a serologic marker of *M. genitalium*, which measures both acute and past exposure. Thus, it may be that only current or recent *M. genitalium* infection is associated with current PID.

The cross-sectional nature of most *M. genitalium* and PID studies has made it difficult to determine whether or not the relationship is causal. However, there are a handful of prospective studies which allow for temporal assessment. Within the PEACH cohort, Haggerty et al. demonstrated that the relationship between *M. genitalium* and endometritis was independent and causal, since among women without concurrent *N. gonorrhoeae* and/or *C. trachomatis*, a positive endometrial PCR test for *M. genitalium* was associated with over a thirteenfold risk of incident endometritis, assessed histologically 30 days following a baseline evaluation of *M. genitalium *(adjusted RR 13.4, 2.4–75.2) [4]. Similarly, in a nested case-control study of 2079 women presenting for pregnancy termination at Malmo University Hospital, *M. genitalium* was significantly associated with postabortal PID (OR 6.3, 95% CI 1.6–25.2) [45]. Lastly, a study of 2378 sexually active female students participating in a chlamydia screening trial in London reported a positive, nonsignificant association between *M. genitalium* and subsequent PID (RR 2.4, 95% CI 0.7–7.5) [46]. There are several reasons why this study's findings are different from those by Haggerty and Bjartling. First, despite the large sample size, the study was underpowered to detect a prospective association between *M. genitalium* and PID. Second, PID was assessed largely by self-report and thus may suffer from misclassification bias. Further, asymptomatic PID could not be captured in this study. Additional prospective studies with active surveillance of PID using biologic markers are needed to fully understand the relationship between *M. genitalium* and PID.

## 4. Does *M. genitalium* Infection Result in Long-Term Reproductive Morbidity?

PID may result in long-term reproductive sequelae, including infertility, ectopic pregnancy, and chronic pelvic pain. Evidence for this comes from the Lund, Sweden cohort study (1960–1984) in which among 2,501 women with clinically suspected PID, salpingitis verified by laparoscopy was associated with infertility, ectopic pregnancy, recurrent PID, and chronic pelvic pain [23, 47]. Additionally, a number of retrospective case-control studies have shown that women with tubal occlusion are more likely to bear chlamydial or gonococcal antibodies, providing human evidence for causal links between chlamydial PID, gonococcal PID, and infertility [48–52]. 

Whether or not *M. genitalium* upper genital tract infection can result in reproductive or gynecologic sequelae is unclear. Like *C. trachomatis*, *M. genitalium* is often asymptomatic [1], increasing the likelihood for “silent” PID and its sequelae. Also parallel to studies of *C. trachomatis*, *M. genitalium* antibodies have been identified more frequently (22% versus 6%) among 132 women with tubal factor infertility compared to 176 women nontubal factor infertility [53]. In a subsequent serologic investigation of *M. genitalium* and tubal factor infertility by the same investigator, 212 couples attending fertility clinics were examined and a strong antibody response against *M. genitalium* or *C. trachomatis*, but no sign of current or chronic infection, was found in women with TFI, indicating that previous infections caused by these microorganisms may have resulted in permanent damage and occlusion of the fallopian tubes [54]. In another study of 51 infertility patients and 23 healthy, fertile women, *M. genitalium* was identified in the cervical canal by PCR among 20% of cases versus 4% of controls (*P* = 0.16) [55]. In subgroup analyses, *M. genitalium *was found in 29% (7 of 24) women with idiopathic infertility, and the comparison to controls was of borderline statistical significance (*P* = 0.05). Although these relationships were not statistically significant, they suggest that current infection with *M. genitalium* and/or permanent damage to the reproductive tract caused by chronic infection with *M. genitalium* may impair fertility. One study has examined the relationship between *M. genitalium* and reproductive morbidity among a population of women with PID. In an analysis of 586 women from the PEACH study presenting with signs and symptoms of PID, Haggerty et al. reported that rates of sequelae, including chronic pelvic pain (42%), infertility (22%), and recurrent PID (31%), were high among women testing positive for active endometrial *M. genitalium* by PCR at baseline [4]. Although differences in rates of sequelae were not significantly different between women testing positive or negative for *M. genitalium*, there was a trend toward increased chronic pelvic pain, infertility, and recurrent PID and decreased pregnancy and live birth following *M. genitalium* infection. The rate of subsequent infertility among women with active endometrial *M. genitalium* was approximately twice as high as the rate reported from a study utilizing the 2002 National Survey of Family Growth data [17], suggesting that preservation of fertility may be suboptimal for women with *M. genitalium* upper genital tract infection. 

Data examining *M. genitalium* and other reproductive consequences are sparse. One serologic case-control study of 82 ectopic pregnancy cases and 246 healthy pregnancy control women found no statistically significant association between ectopic pregnancy and *M. genitalium* antibodies [44]. Nonsignificant trends suggesting an association between *M. genitalium* and ectopic pregnancy were found among a subgroup of women aged 18–30 (OR 2.0, *P* = 0.133) and among women testing negative for *C. trachomatis* antibodies (OR 2.3, *P* = 0.161) [44]. It may be possible that reduced power in these subset analyses limited the ability to detect statistically significant associations. Further large prospective studies utilizing both serology and PCR are needed to better understand the potential reproductive sequelae of *M. genitalium* infection.

## 5. Symptoms of *M. genitalium* and Implications for Delayed Treatment

Although some studies have linked *M. genitalium* to pathologic vaginal discharge [56] and urethritis [57], several have reported that both *M. genitalium* [7, 58, 59] and *C. trachomatis* [60] are comparatively less symptomatic than gonococcal infection [60]. Harboring an asymptomatic infection may increase the likelihood for delayed care and development of sequelae. In a study of 516 sexual dyads, although *M. genitalium* was associated with urethral discharge in men, no symptoms were diagnostic of infection in women [8]. In addition, *M. genitalium* was found to be common in asymptomatic patients attending an STD clinic in the United Kingdom [61]. 

Symptoms of PID vary by microbial pathogen. For example, chlamydial salpingitis tends to exhibit more mild symptoms than gonococcal PID, despite the fact that both pathogens cause tubal damage [60]. Short et al. found that, compared to women with gonococcal PID, those with *M. genitalium*-associated PID were less likely to have elevated markers of inflammation, cervicitis, elevated vaginal pH, and a high pelvic pain score [58]. However, signs and symptoms of PID were similar between women with *C. trachomatis* and *M. genitalium* [58]. This may indicate that, among women with PID, those infected with *N. gonorrhoeae* present with more overt and severe symptoms, leading to earlier treatment than women with *C. trachomatis *or *M. genitalium* [60]. Long time to treatment is a major concern, as a case-control study nested within a landmark Scandinavian study found that delaying care for 3 or more days significantly increased the risk of impaired fertility among 443 women with PID [62]. In a more recent study of 298 women with histologically confirmed endometritis, those with *C. trachomatis* monoinfection and *M. genitalium* monoinfection reported waiting the longest time between onset of symptoms and care seeking (12.3 and 10.9 days), while the shortest times were among women with *N. gonorrhoeae* monoinfection (4.6 days) and coinfection with two or more pathogens (5.6 days) [63]. Delayed treatment of PID for 14 days or more was not significantly associated with reproductive morbidity in this study. However, rates of infertility, recurrent PID, and chronic pelvic pain were high in this cohort (17%, 20%, and 36%). Collectively, these studies may suggest that women with *M. genitalium*-associated PID may have low levels of chronic inflammation that can lead to reproductive damage before treatment.

## 6. Treatment of Upper Genital Tract *M. genitalium* Infection

If women with *M. genitalium* upper genital tract infection do seek care, they will likely be treated with one of the currently recommended CDC treatment regimens for PID including (1) ofloxacin, (2) levofloxacin, (3) ceftriaxone plus doxycycline, or (4) cefoxitin and probenecid plus doxycycline; all with optional metronidazole for full coverage against anaerobes and BV [64]. However, some of these regimens are ineffective for the treatment of *M. genitalium*. In the PEACH study, Haggerty et al reported that persistence of *M. genitalium* was very high among women treated with cefoxitin and doxycycline for PID, with 44% of women with baseline endometrial PCR-positive specimens testing positive again 30 days following treatment [4]. In contrast, only 2% to 4% of women in the PEACH study had persistent or recurrent gonococcal or chlamydial cervicitis when retested at 30 days [24]. Women with *M. genitalium* identified in the endometrium by PCR at study enrollment were four times as likely to experience persistent endometritis and over four times as likely to experience treatment failure, defined as the presence of both endometritis and pelvic pain 30 days following treatment for PID (adjusted RR 4.6, 95% CI 1.1–20.1) [4]. Further, *M. genitalium* strains resistant to tetracycline have been isolated [65], and *M. genitalium* is associated with persistent nongonococcal urethritis among men treated with tetracyclines [35, 59, 66–68] and levofloxacin [69, 70] for nongonococcal urethritis. Thus, even if women with active *M. genitalium* upper genital tract infection seek treatment, antibiotic resistance among *M. genitalium* strains may lead to persistent or recurrent infection, resulting in chronic inflammation and infection.

## 7. Conclusion

PID is a common disease among American women that results in frequent, serious reproductive morbidity. Most women with PID are treated with antibiotics directed toward *N. gonorrhoeae* and/or *C. trachomatis*, despite the fact that these bacterial pathogens account for only a third to a half of PID cases. Although *M. genitalium* has recently been recognized as a cause of nongonococcal, nonchlamydial PID, little is known about the long-term prognosis of *M. genitalium *upper genital tract infection. 

Given the scarcity of information regarding the long-term prognosis of women infected with *M. genitalium*, the lack of routine testing for *M. genitalium* in clinical practice, and the resistance of *M. genitalium* to a number of PID treatment regimens, additional research on the relationships between *M. genitalium*, PID, and long-term reproductive sequelae is critically needed in order to shape screening and treatment guidelines. The high rate of treatment failure among women with clinically suspected PID testing positive for *M. genitalium* emphasizes a need for PID antibiotic regimens targeted toward *M. genitalium*, with the ultimate goal to prevent reproductive and gynecologic morbidity. *M. genitalium* has demonstrated susceptibility to macrolides, with azithromycin being the most active, and variable resistance to fluroquinolones, including ciprofloxacin [36, 71]. However, it should be noted that *M. genitalium* azithromycin resistance has recently been reported [72, 73]. A newer quinolone, moxiflocacin, has recently been shown to exhibit a high degree of activity against *M. genitalium* [74], and this antibiotic has also been shown to be effective for the treatment of PID [75]. Although these promising therapies warrant further study for the treatment of PID, no highly sensitive test is widely used to diagnose *M. genitalium* in clinical practice. Nucleic acid amplification tests (NAATs) have been developed and tested [76], and they may be useful for the clinical detection of *M. genitalium* among PID patients. Endocervical swabs collected from patients with clinically suspected PID are already often tested for gonococcal and chlamydial infection, and thus a NAAT for *M. genitalium* could efficiently be added to this diagnostic screening. *M. genitalium* screening among patients with clinically suspected PID would allow clinicians to select treatment regimens specific for mycoplasmal PID. Additionally, commercially available testing is also critical for the identification and treatment of uncomplicated lower genital tract *M. genitalium* infection, in order to prevent subsequent PID and potential sequelae. 

## Figures and Tables

**Table 1:** Studies evaluating the relationship between *M. genitalium* and pelvic inflammatory disease.

Citation	Sample size, population, setting	Study design	Methods: *M. genitalium* test PID diagnosis	Findings	Validity
Uno et al. [13]	200 patients aged 19 to 49 years visiting the OB Gyn department of Kizawa Memorial Hospital and Hayasaki Ladies Clinic.	Cross-sectional	*M. genitalium*: PCR of endocervical specimens. PID: clinical criteria.	5.7% of PID patients versus 0% of pregnant controls tested positive for *M. genitalium* (*P*-value or OR not reported, one patient co-infected with *C. trachomatis*).	Strengths: Control group of patients without signs and symptoms of PID. Limitations: No laparoscopic or histologic confirmation of PID. Although *C. trachomatis* was assessed, sample size too small to determine independent effect of *M. genitalium *

Cohen et al. [6]	115 patients presenting with pelvic pain ≤ 14 days presenting to a sexually transmitted diseases clinic, Nairobi, Kenya between 2000–2003.	Cross-sectional	*M. genitalium: *PCR of cervical and endometrial samples. PID**:** histologically confirmed endometritis.	*M. genitalium *detected in 16% of patients with endometritis versus 2% of patients without endometritis (*P* = 0.03). *M. genitalium* identified in the endometrium was associated with endometritis after excluding women with gonococcal or chlamydial infection (*P* = 0.03, percentages not presented in the paper).	Strengths: PID defined histologically. Adjustment for *C. trachomatis* and *N. gonorrhoeae *allows for independent association between *M. genitalium* and PID to be examined. Limitations: No control group of women without clinically suspected PID. Cross-sectional design does not allow for a temporal association to be proven

Simms et al. [43]	45 patients with a clinical diagnosis of PID (ages 16–43) and 37 patients undergoing tubal ligation (ages 21–45).	Case-control study	*M. genitalium: *PCR of endocervical swabs. PID: clinical criteria.	*M. genitalium *detected in 13% of patients versus 0% of controls.	Strengths: Control group of patients without signs and symptoms of PID (although not confirmed histologically or laparoscopically. Limitations: No upper genital tract specimens collected. PID was not confirmed laparoscopically or histologically. No adjustment for confounders. Cross-sectional design does not allow for a temporal association to be proven.

Cohen et al. [41]	123 women aged 18–40 with laparoscopically confirmed PID treated at Kenyatta National Hospital, 2000–2003.	Cross-sectional study	*M. genitalium*: PCR of cervical, endometrial, and fallopian tube samples. PID: laparoscopically diagnosed salpingitis, graded as mild, moderate, or severe.	*M. genitalium* detected in the fallopian tube of one patient. 6% of women with mild, 11% of women with moderate, and 6% of women with severe salpingitis tested positive for *M. genitalium* in one or more site.	Strengths: PID verified by laparoscopy. Limitations: No control group of women without PID. Cross-sectional design does not allow for a temporal association to be proven.

Jurstrand et al. [44]	194 inpatients with PID aged 15–50 and 83 inpatients with ectopic pregnancy (EP) aged 18–42 treated in the OBGyn department of Örebo University Hospital, Örebo, Sweden, 1984–1986. 246 healthy pregnant women being screened for rubella were matched to ectopic pregnancy cases by age.	Case control study	*M. genitalium*: antibodies assessed using a lipid-associated membrane protein-enzyme immunoassay (LAMP-EIA). PID: clinical criteria.	*M. genitalium* and PID: Crude OR 1.3 (0.7–2.2). Adjusted OR 1.0 (0.6–1.7) *M. genitalium* & EP: Crude OR 1.3 (0.7–2.5). Adjusted OR 1.0 (0.5–2.0). ^(^Adjusted for age and *C. trachomatis* antibodies.)	Strengths: LAMP-EIA covers antigenic variation of different genotypes of *M. genitalium* with no cross-reactivity with other Mycoplasma species. Limitations: PID not laparoscopically or histologically confirmed. Limited adjustment for confounders. Unable to determine timing of *M. genitalium* infection in relation to the acute PID episode.

Haggerty et al. [4]	682 women with clinically suspected PID aged 14–37 years recruited from ER, OB/Gyn, STD clinics, and private practice from 13 U.S. urban clinical sites, 1996–1999.	Prospective	*M. genitalium:* PCR of cervical and endometrial samples. PID: histologically confirmed endometritis assessed at baseline and at a 30-day follow-up clinic visit.	Baseline comparison of *M. genitalium* (endometrium) and endometritis: Adjusted OR 3.0 (1.5–6.1). Prospective evaluation of baseline *M. genitalium *and incident endometritis (30-days follow-up visit): Adjusted RR 13.4 (2.4–75.2). ^(^Adjusted for age, race, *C. trachomatis*, and *N. gonorrhoeae.*)	Strengths: Large sample size. Histologic confirmation of PID. Prospective analysis of baseline *M. genitalium* infection and incident endometritis at the 30 day follow-up visit supports a temporal association. Adjustment for *C. trachomatis* and *N. gonorrhoeae *allows for independent association between *M. genitalium* and PID to be examined. Limitations: No control group of women without clinically suspected PID

Bjartling et al. [45]	2079 women aged 15–40 presenting for termination of pregnancy at Malmö University Hospital, Sweden, 2003–2007.	Prospective	*M. genitalium:* PCR of urine, vaginal, and cervical samples. PID: clinical criteria.	*M. genitalium* & posttermination PID: Adjusted OR 6.3 (1.6–25.2). ^(^Adjusted for age and *C. trachomatis.*)	Strengths: Prospective design allows for temporal inference. Adjustment for *C. trachomatis* allows for independent association between *M. genitalium* and PID to be examined. Limitations: Generalizability limited to post-abortal PID. No upper genital tract specimens tested. PID not confirmed laparoscopically or histologically.

Oakeshott et al. [46]	2378 sexually active female students (mean age 21 years) participating in a chlamydia screening trial, London, 2004–2006.	Prospective	*M. genitalium*: PCR of self-collected vaginal swabs. PID: Self-reported PID and PID symptoms, and medical records/clinical diagnosis for a subset of women not completing follow-up questionnaire, assessed over 12 months.	*M. genitalium* & PID: RR 2.4 (0.7–7.5).	Strengths. Large sample size. Prospective design allows a temporal relationship to be explored. Limitations: PID diagnosis based on self-report and limited medical record/clinical diagnosis; no laparoscopic or histologic confirmation. Asymptomatic PID not captured. Despite large sample, the study was underpowered to detect a prospective association between *M. genitalium* and PID. Selection bias may have caused an underestimate of *M. genitalium. *
